# Supplementary material for: Synthesis, Anti-Proliferative Evaluation and Mechanism of 4-Trifluoro Methoxy Proguanil Derivatives with Various Carbon Chain Length
Source: Molecules. 2021 Sep 24;26(19):5775. doi: 10.3390/molecules26195775 (PMC8510509; doi:10.3390/molecules26195775)
Supplement: Supplementary file 1 [file molecules-26-05775-s001.zip › molecules-1363349-supplementary.pdf]

Supplemental Information

Synthesis, anti-proliferative evaluation and mechanism of 4-trifluoro methoxy proguanil derivatives with various carbon chain length

Simeng Xu[1,#], Yufang Cao[1,#], Yu Luo[2], Di Xiao[1], Wei Wang[2], Zhiren Wang[1] and Xiaoping Yang[1,*]

1 Key Laboratory of Study and Discovery of Small Targeted Molecules of Hunan Province, Key Laboratory of Protein Chemistry and Developmental Biology of Fish of Ministry of Education, Department of Pharmacy, School of Medicine, Hunan Normal University, Changsha, Hunan, China.

2 TCM and Ethnomedicine Innovation & Development International Laboratory, Innovative Material Medical Research Institute, School of Pharmacy, Hunan University of Chinese Medicine, Changsha, Hunan, China

# These authors made the same contribution to this article

*Corresponding author

Tel/Fax: 01186-158-7406-6132

E-mail address：xiaoping.yang@hunnu.edu.cn (X. Yang)

Figure S1. NMR spectra of target derivatives

2C

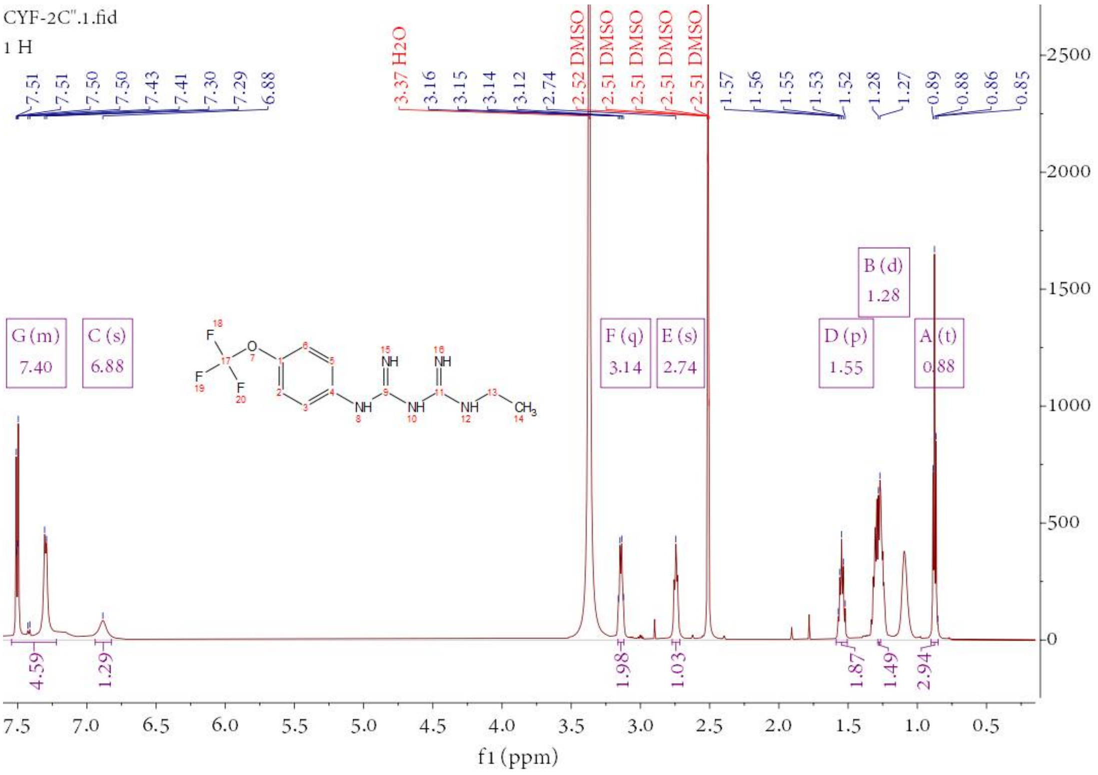

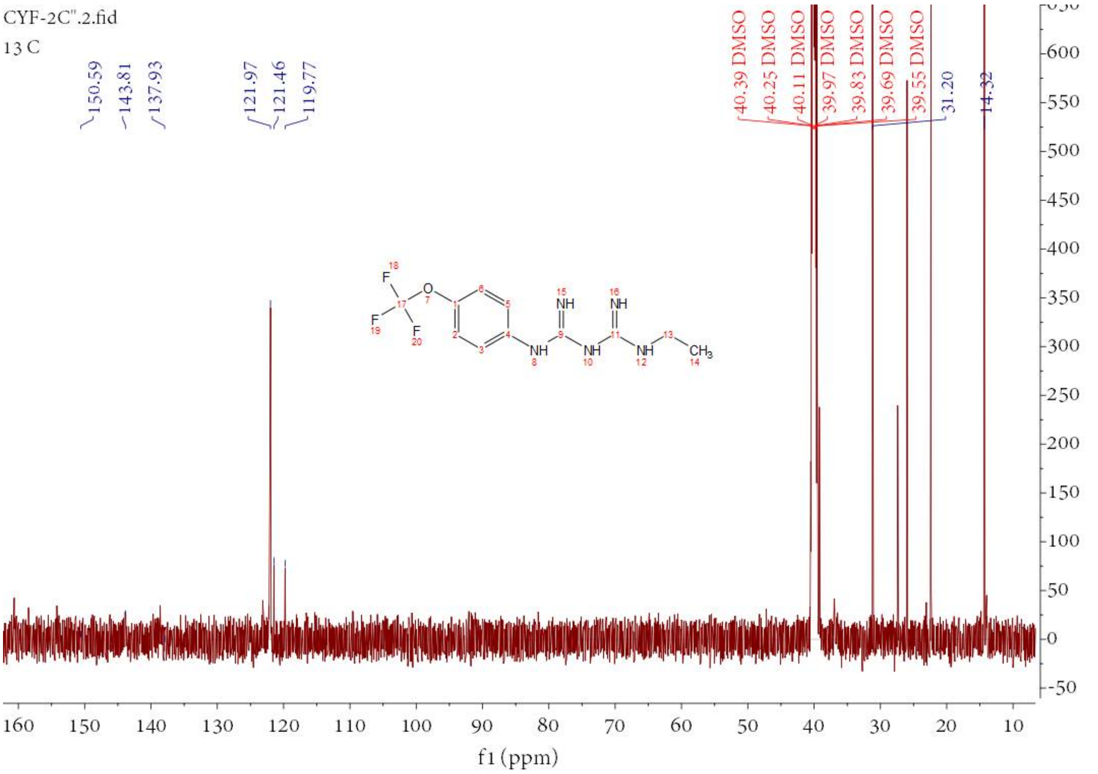

3C

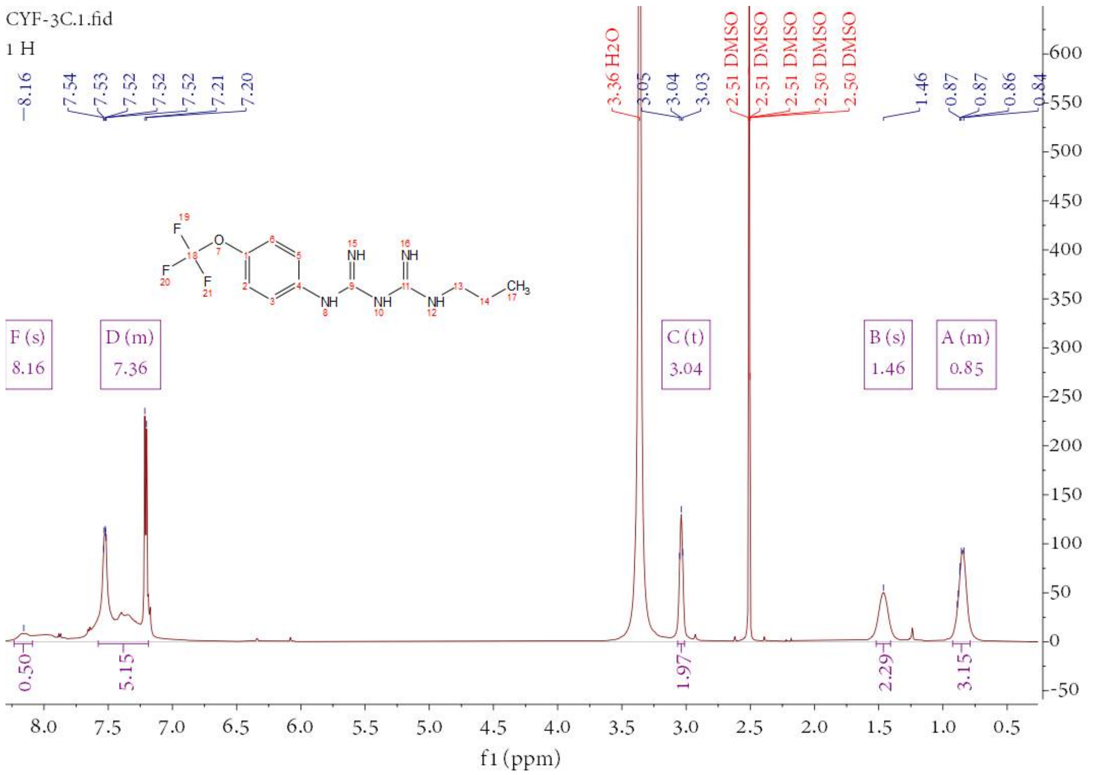
CYF-3C.1.fid
1 H
8.16
7.54
7.53
7.52
7.52
7.52
7.21
7.20
3.36 H2O
3.05
3.04
3.03
2.51 DMSO
2.51 DMSO
2.51 DMSO
2.50 DMSO
2.50 DMSO
1.46
0.87
0.87
0.86
0.84
F (s) 8.16
D (m) 7.36
C (t) 3.04
B (s) 1.46
A (m) 0.85
0.50
5.15
1.97
2.29
3.15
f1 (ppm)

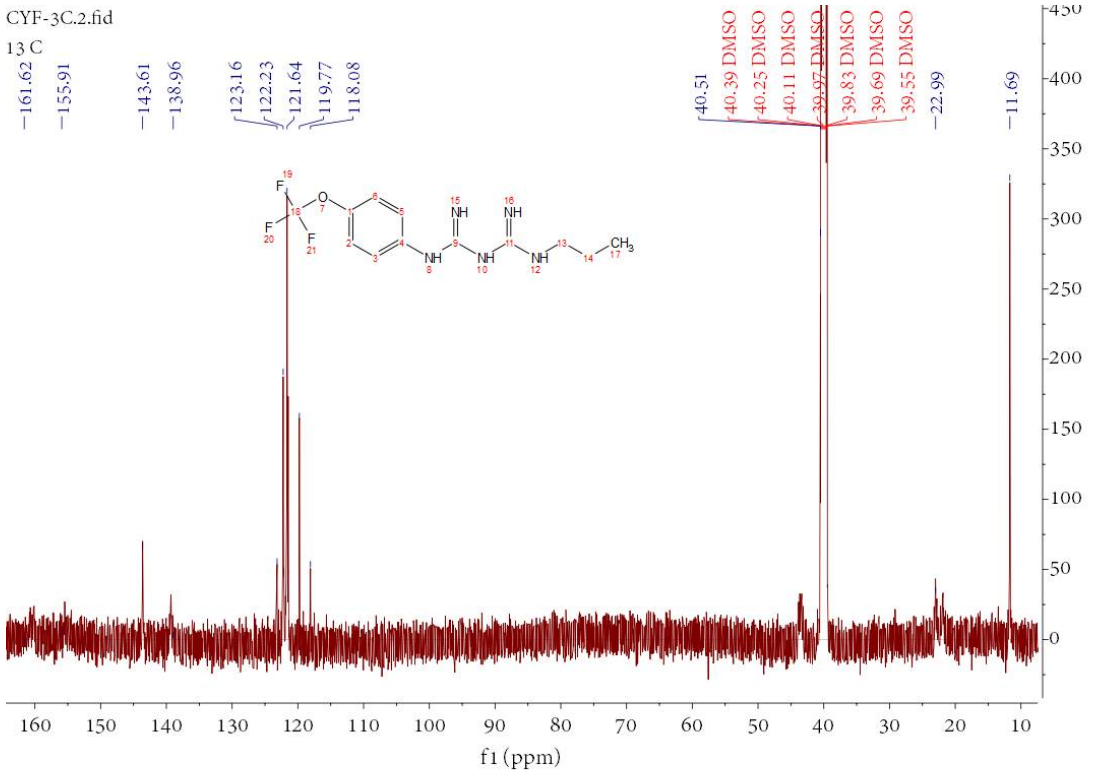
CYF-3C.2.fid
13 C
161.62
155.91
143.61
138.96
123.16
122.23
121.64
119.77
118.08
40.51
40.39 DMSO
40.25 DMSO
40.11 DMSO
39.97 DMSO
39.83 DMSO
39.69 DMSO
39.55 DMSO
22.99
11.69
f1 (ppm)

4C

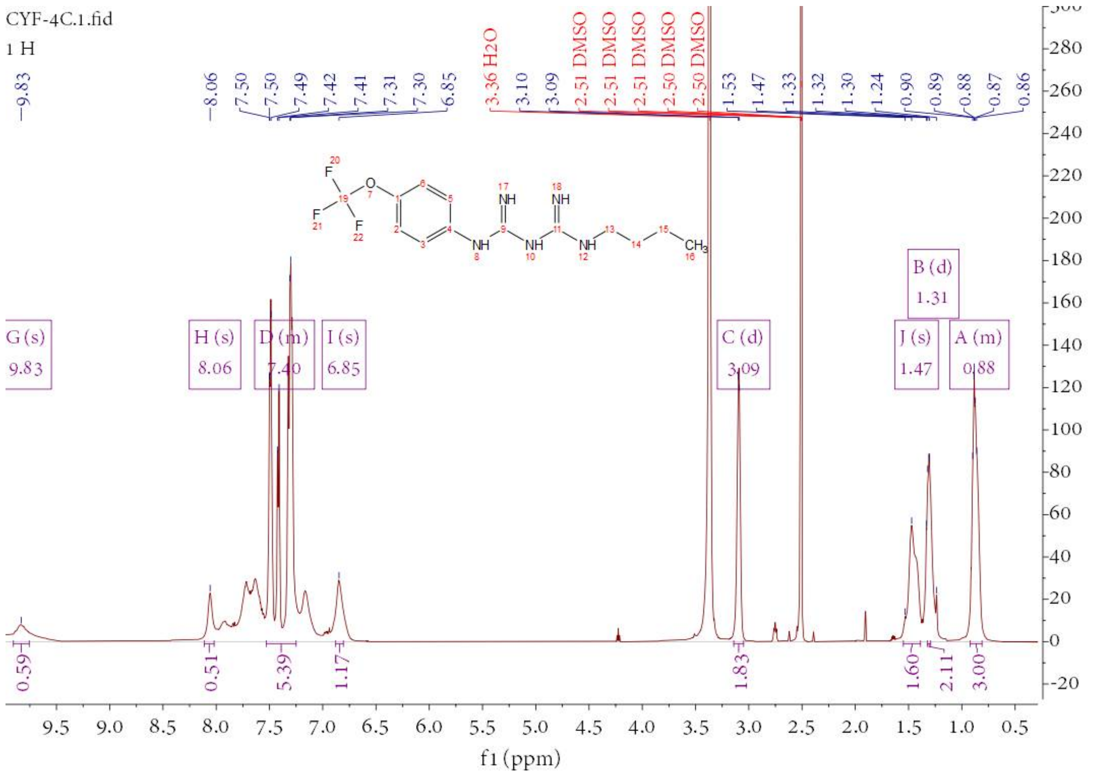
CYF-4C.1.fid
1 H
9.83
8.06
7.50
7.50
7.49
7.42
7.41
7.31
7.30
6.85
3.36 H2O
3.10
3.09
2.51 DMSO
2.51 DMSO
2.51 DMSO
2.50 DMSO
2.50 DMSO
1.53
1.47
1.33
1.32
1.30
1.24
0.90
0.89
0.88
0.87
0.86
G (s)
9.83
H (s)
8.06
D (m)
7.40
I (s)
6.85
C (d)
3.09
B (d)
1.31
J (s)
1.47
A (m)
0.88
0.59
0.51
5.39
1.17
1.83
1.60
2.11
3.00
f1 (ppm)

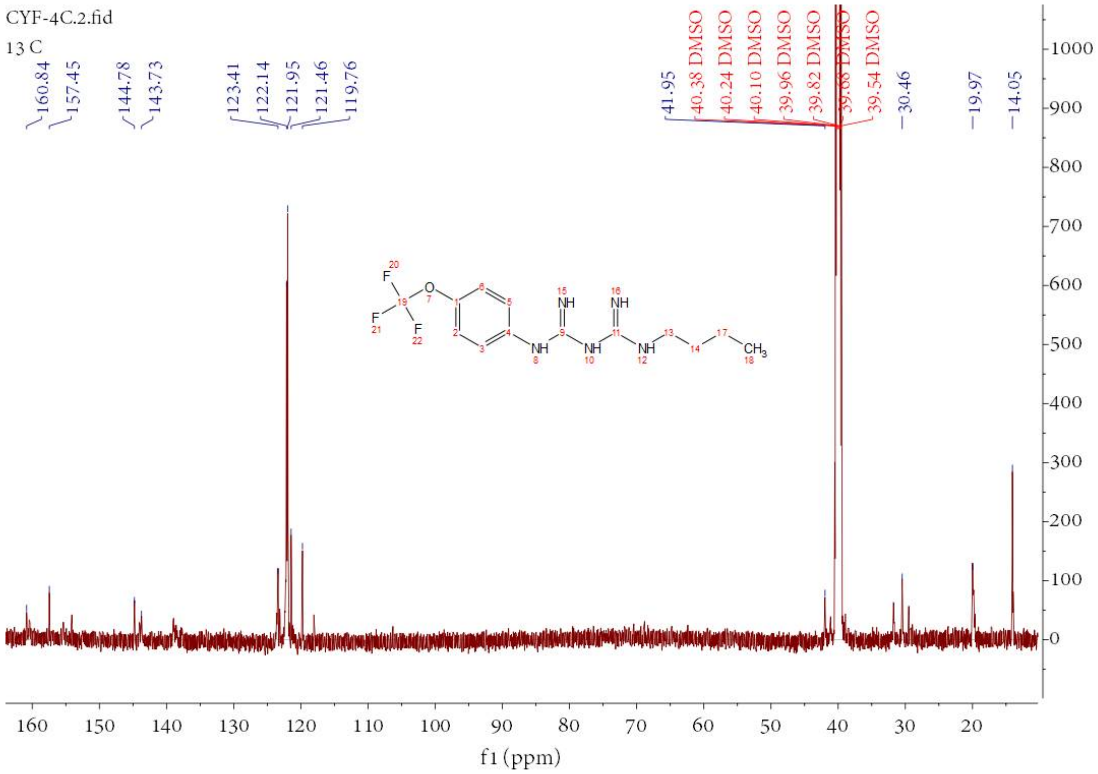
CYF-4C.2.fid
13 C
160.84
157.45
144.78
143.73
123.41
122.14
121.95
121.46
119.76
41.95
40.38 DMSO
40.24 DMSO
40.10 DMSO
39.96 DMSO
39.82 DMSO
39.68 DMSO
39.54 DMSO
30.46
19.97
14.05
f1 (ppm)

5C

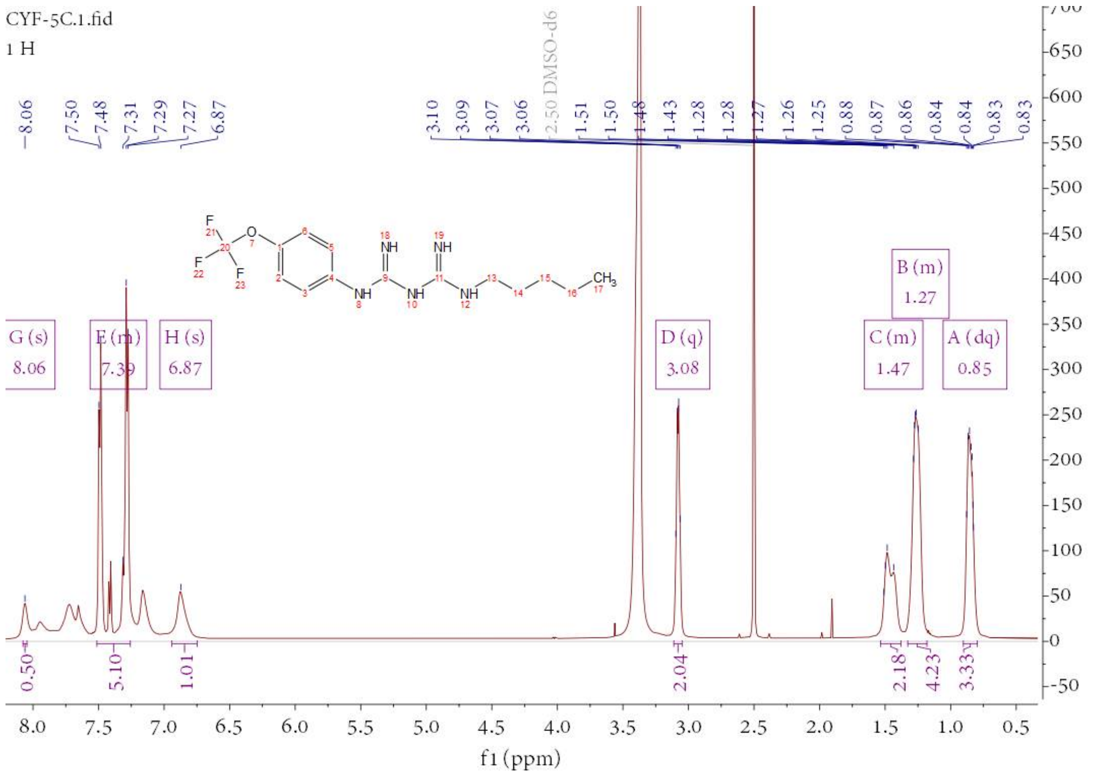
CYF-5C.1.fid
1 H
G (s) 8.06
E (m) 7.39
H (s) 6.87
D (q) 3.08
B (m) 1.27
C (m) 1.47
A (dq) 0.85
2.50 DMSO-d6
f1 (ppm)

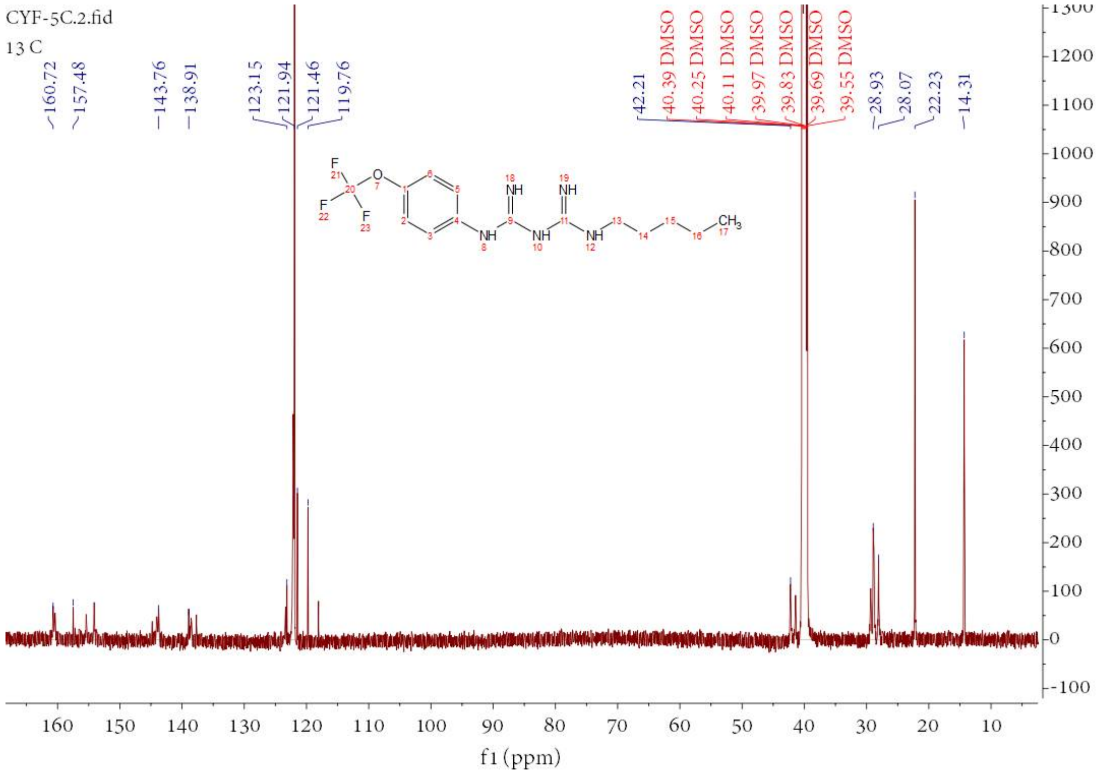
CYF-5C.2.fid
13 C
160.72
157.48
143.76
138.91
123.15
121.94
121.46
119.76
42.21
40.39 DMSO
40.25 DMSO
40.11 DMSO
39.97 DMSO
39.83 DMSO
39.69 DMSO
39.55 DMSO
28.93
28.07
22.23
14.31
f1 (ppm)

6C

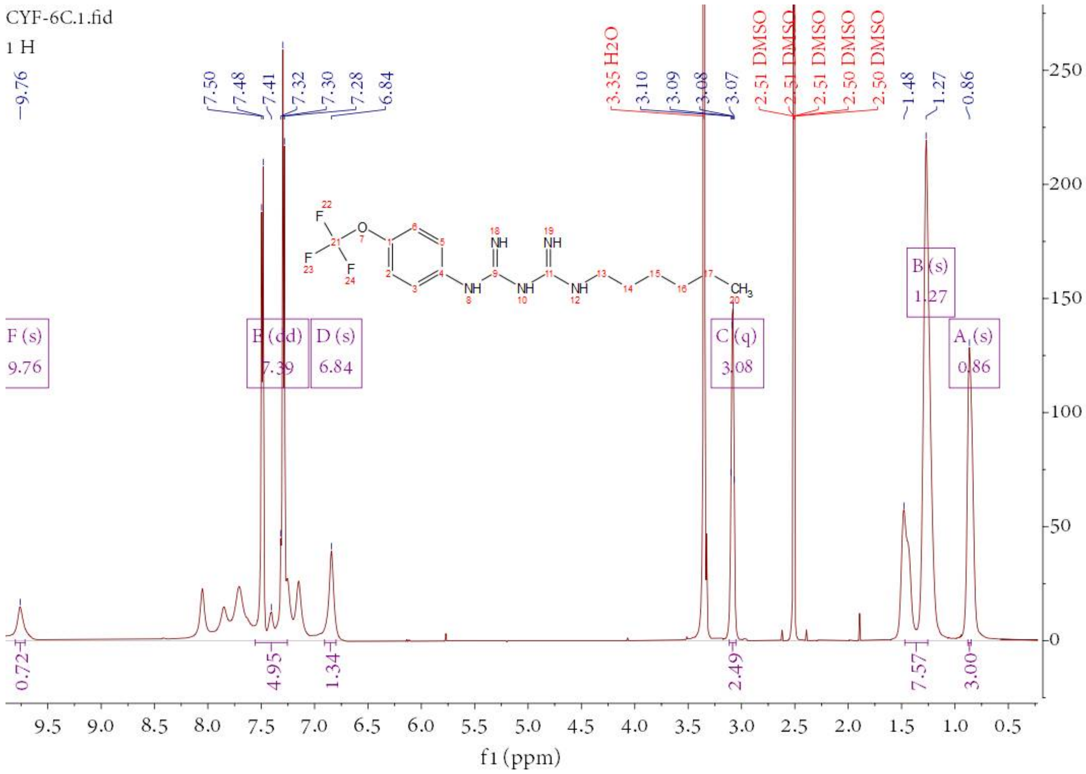
CYF-6C.1.fid
1 H
9.76
7.50 7.48 7.41 7.32 7.30 7.28 6.84
3.35 H2O
3.10 3.09 3.08 3.07
2.51 DMSO 2.51 DMSO 2.51 DMSO 2.50 DMSO 2.50 DMSO
1.48 1.27 0.86
F (s) 9.76
E (dd) 7.39
D (s) 6.84
C (q) 3.08
B (s) 1.27
A (s) 0.86
0.72 4.95 1.34 2.49 7.57 3.00
f1 (ppm)

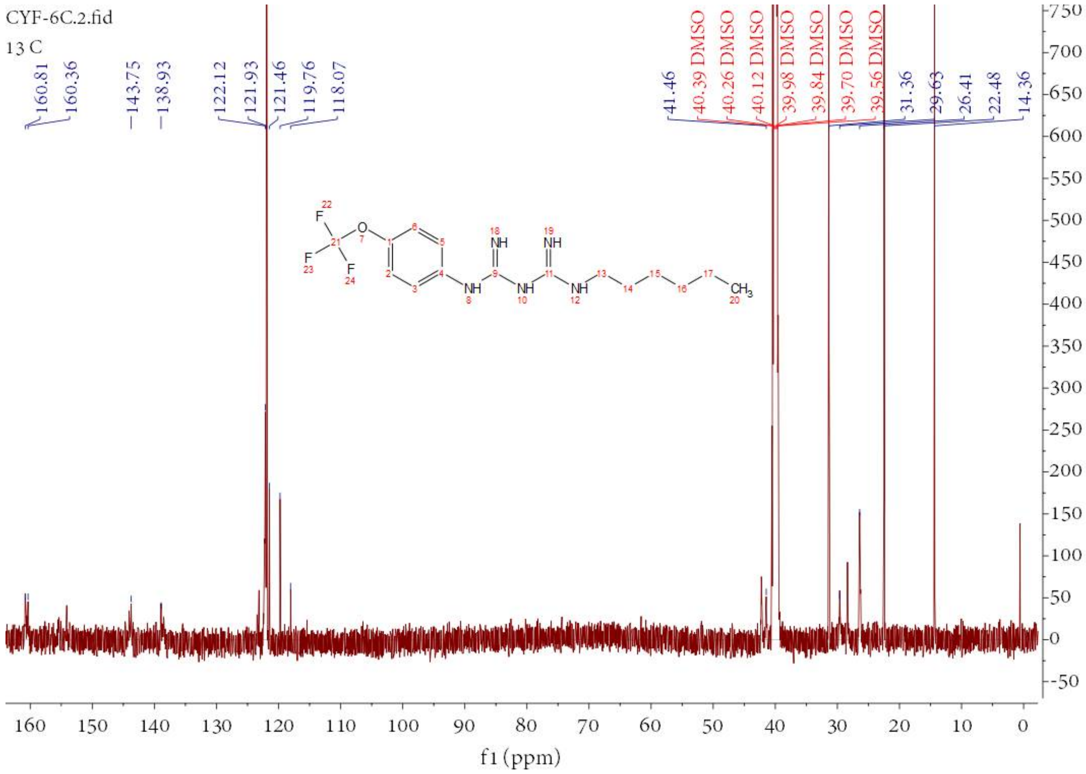
CYF-6C.2.fid
13 C
160.81 160.36 143.75 138.93 122.12 121.93 121.46 119.76 118.07
41.46
40.39 DMSO 40.26 DMSO 40.12 DMSO 39.98 DMSO 39.84 DMSO 39.70 DMSO 39.56 DMSO
31.36 29.63 26.41 22.48 14.36
f1 (ppm)

7C

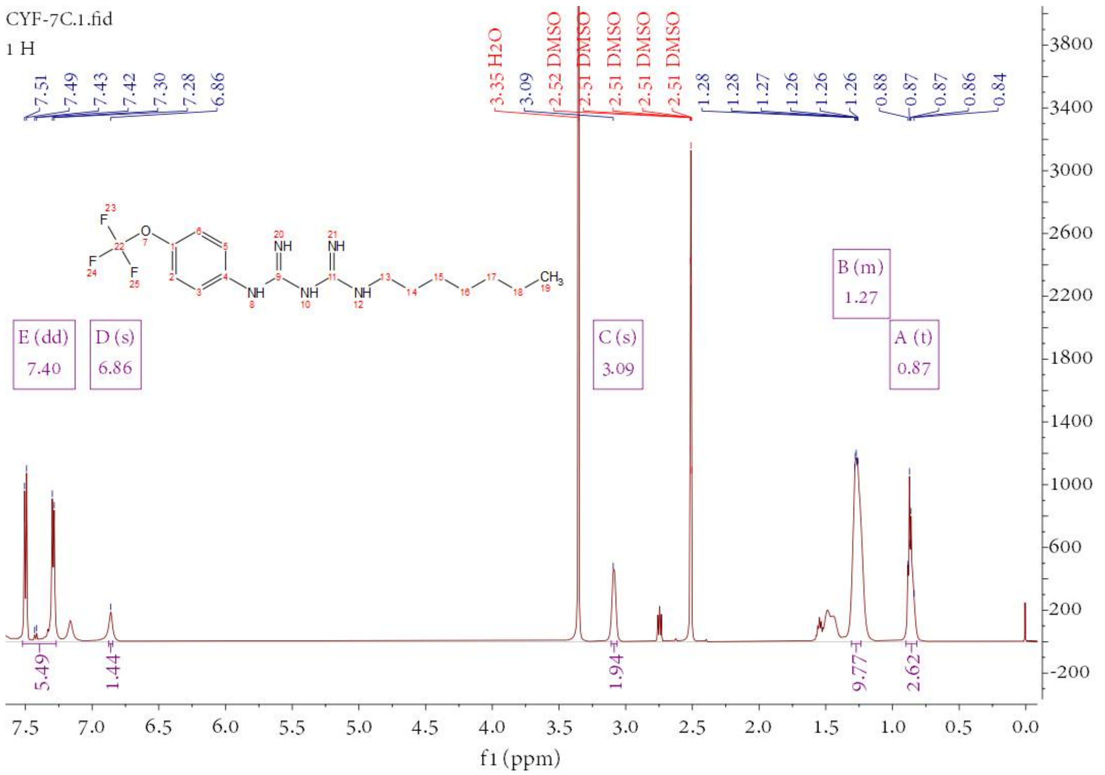

CYF-7C.1.fid
1 H
7.51
7.49
7.43
7.42
7.30
7.28
6.86
3.35 H2O
3.09
2.52 DMSO
2.51 DMSO
2.51 DMSO
2.51 DMSO
2.51 DMSO
1.28
1.28
1.27
1.26
1.26
1.26
0.88
0.87
0.87
0.86
0.84
E (dd)
7.40
D (s)
6.86
C (s)
3.09
B (m)
1.27
A (t)
0.87
5.49
1.44
1.94
9.77
2.62
f1 (ppm)

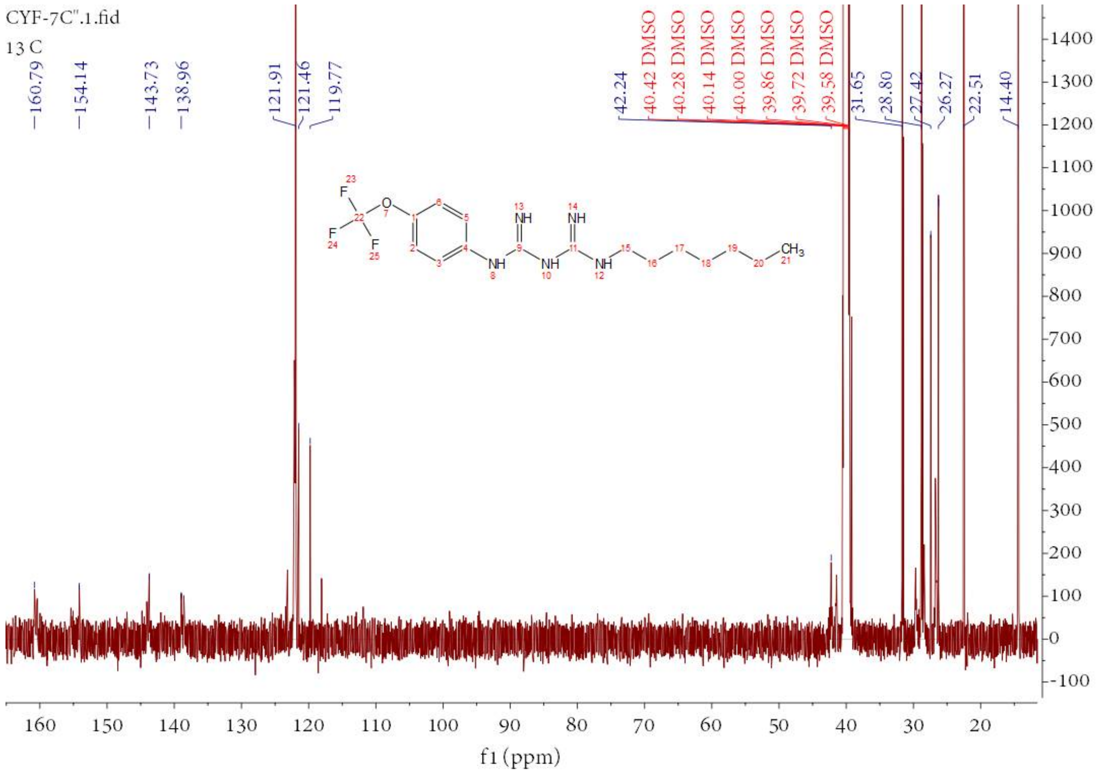

CYF-7C".1.fid
13 C
160.79
154.14
143.73
138.96
121.91
121.46
119.77
42.24
40.42 DMSO
40.28 DMSO
40.14 DMSO
40.00 DMSO
39.86 DMSO
39.72 DMSO
39.58 DMSO
31.65
28.80
27.42
26.27
22.51
14.40
f1 (ppm)

8C

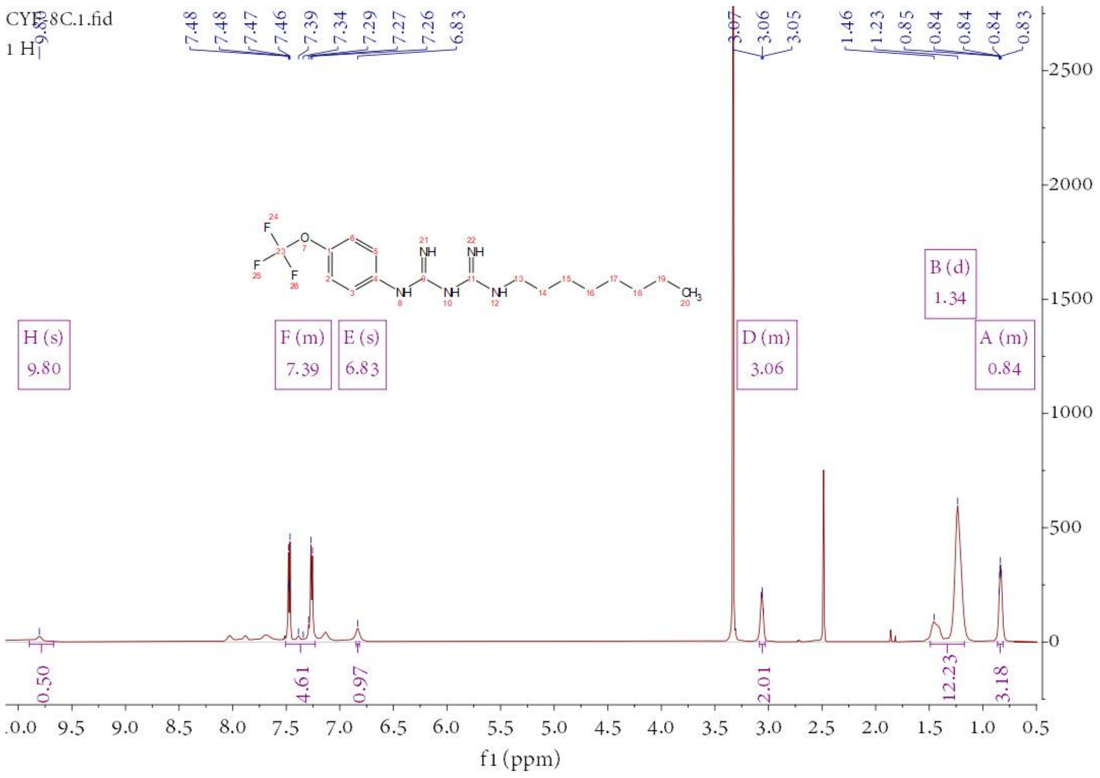

CYF-8C.1.fid
1 H
H (s) 9.80
F (m) 7.39
E (s) 6.83
D (m) 3.06
B (d) 1.34
A (m) 0.84
f1 (ppm)

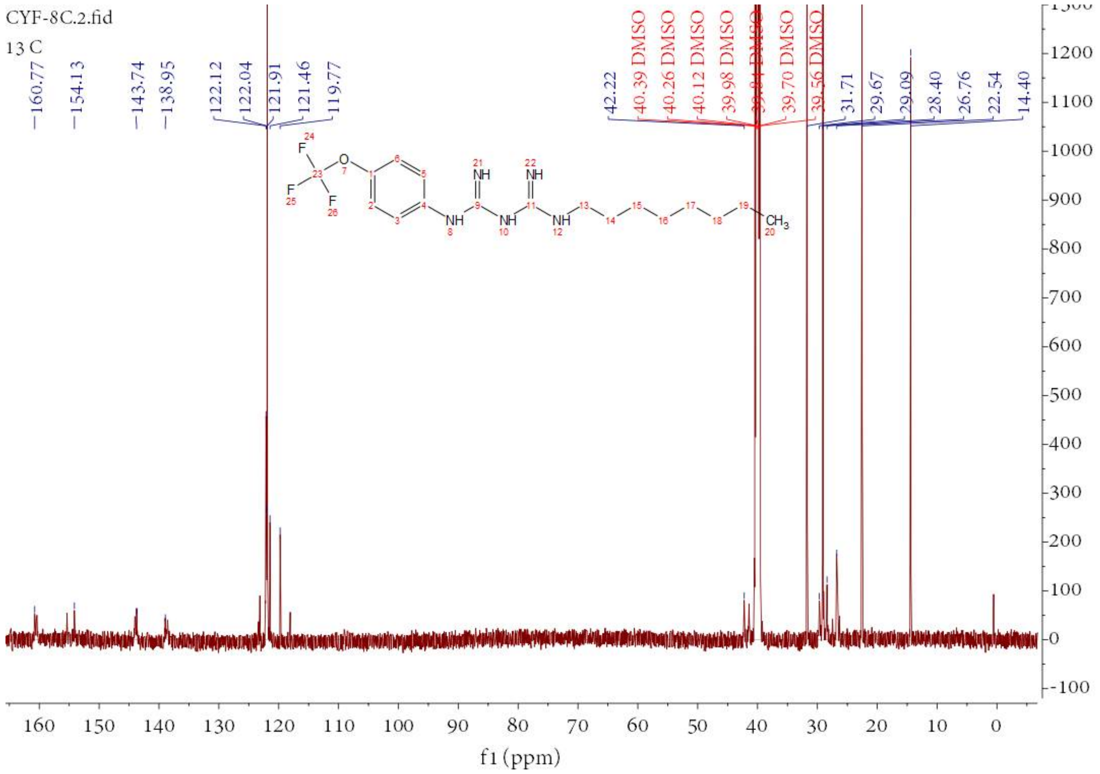

CYF-8C.2.fid
13 C
160.77
154.13
143.74
138.95
122.12
122.04
121.91
121.46
119.77
42.22
40.39 DMSO
40.26 DMSO
40.12 DMSO
39.98 DMSO
39.70 DMSO
39.56 DMSO
31.71
29.67
29.09
28.40
26.76
22.54
14.40
f1 (ppm)

9C

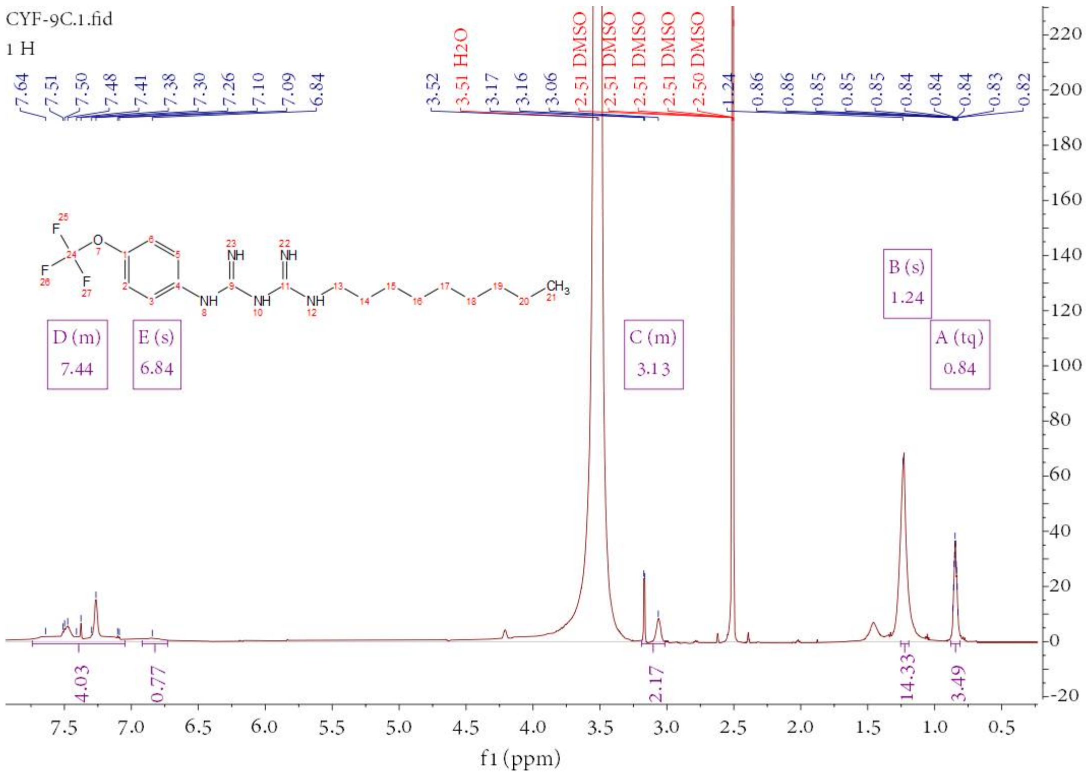

CYF-9C.1.fid
1 H
7.64
7.51
7.50
7.48
7.41
7.38
7.30
7.26
7.10
7.09
6.84
3.52
3.51 H2O
3.17
3.16
3.06
2.51 DMSO
2.51 DMSO
2.51 DMSO
2.51 DMSO
2.50 DMSO
1.24
0.86
0.86
0.85
0.85
0.85
0.84
0.84
0.84
0.83
0.82
D (m)
7.44
E (s)
6.84
C (m)
3.13
B (s)
1.24
A (tq)
0.84
4.03
0.77
2.17
14.33
3.49
f1 (ppm)

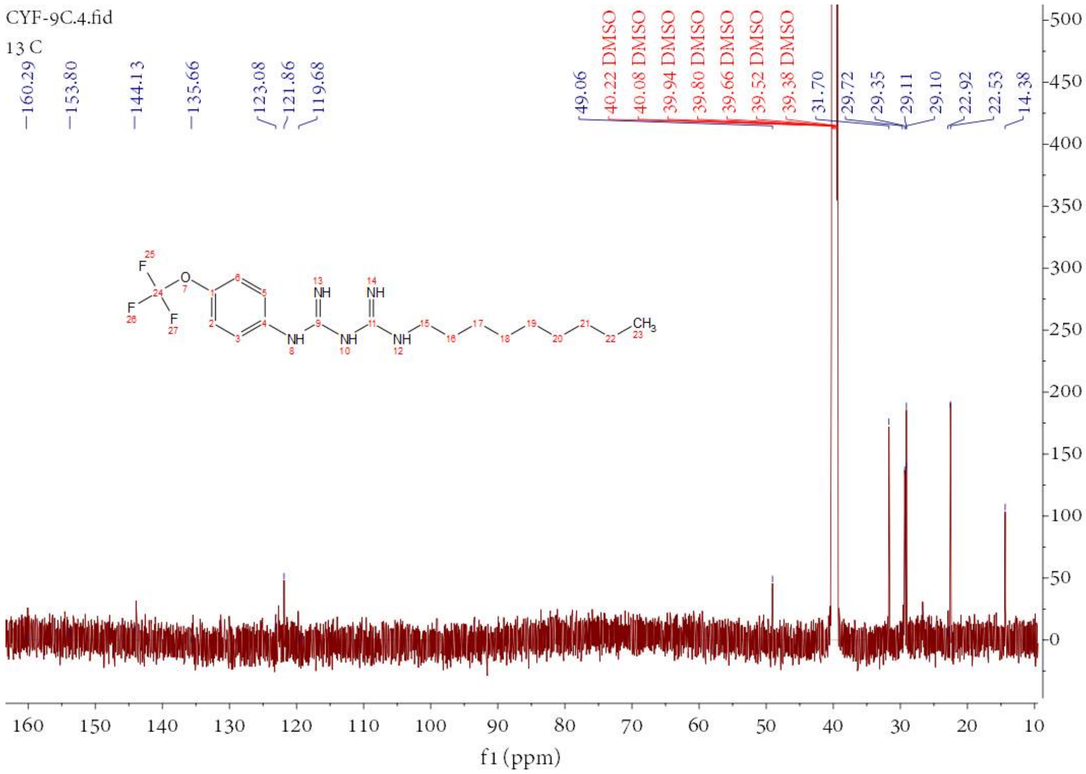

CYF-9C.4.fid
13 C
160.29
153.80
144.13
135.66
123.08
121.86
119.68
49.06
40.22 DMSO
40.08 DMSO
39.94 DMSO
39.80 DMSO
39.66 DMSO
39.52 DMSO
39.38 DMSO
31.70
29.72
29.35
29.11
29.10
22.92
22.53
14.38
f1 (ppm)

10C

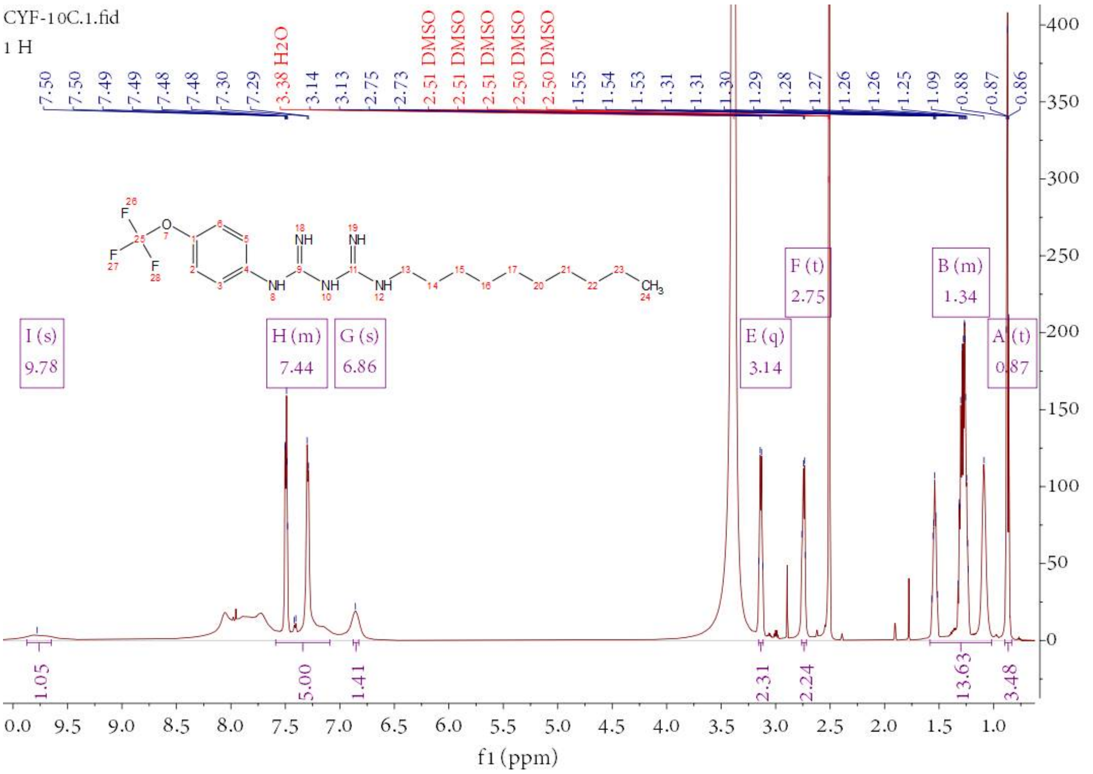

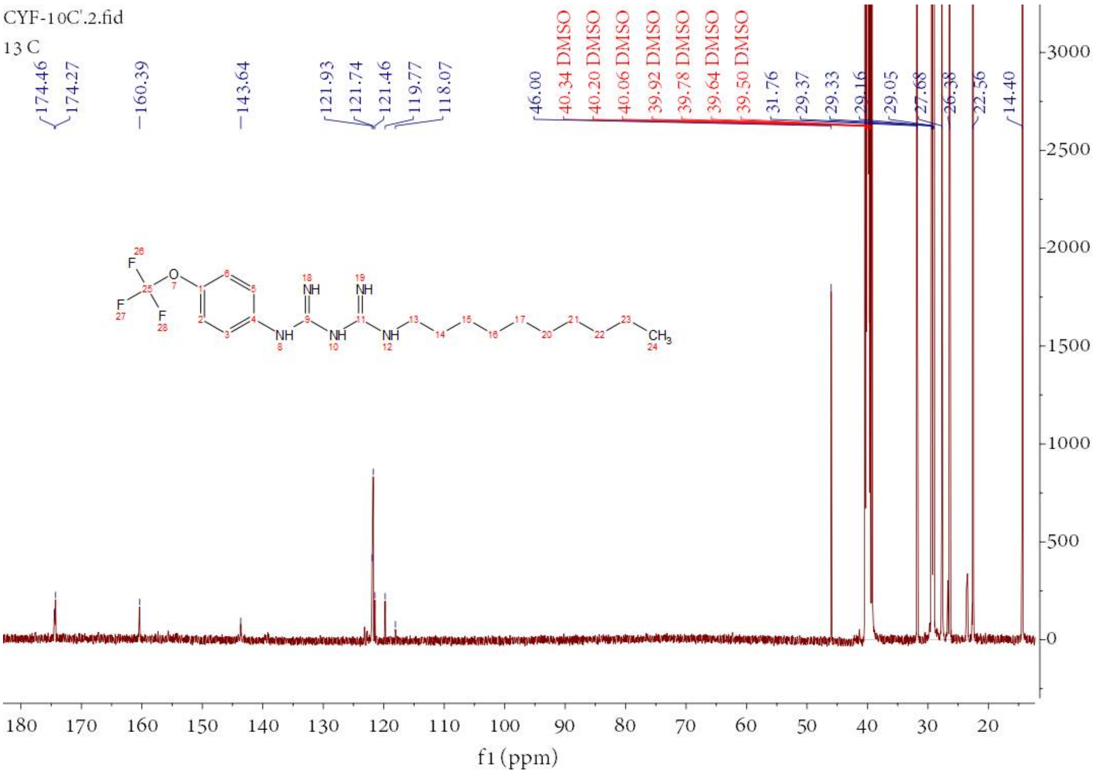

12C

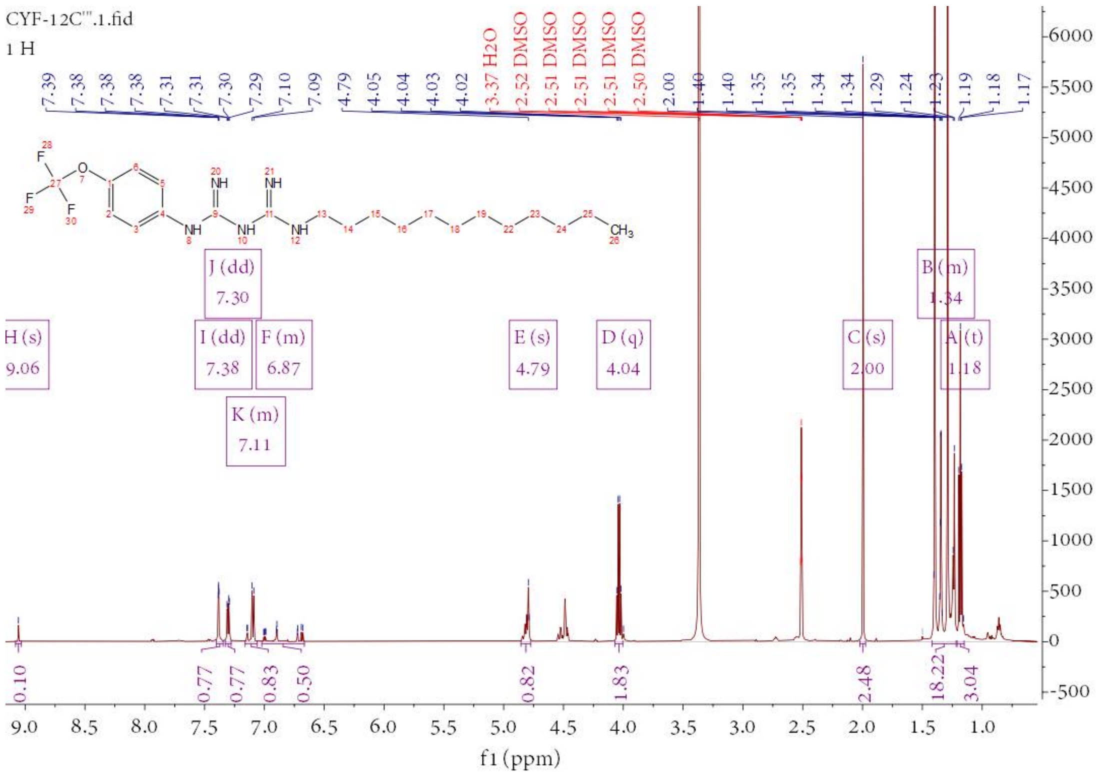

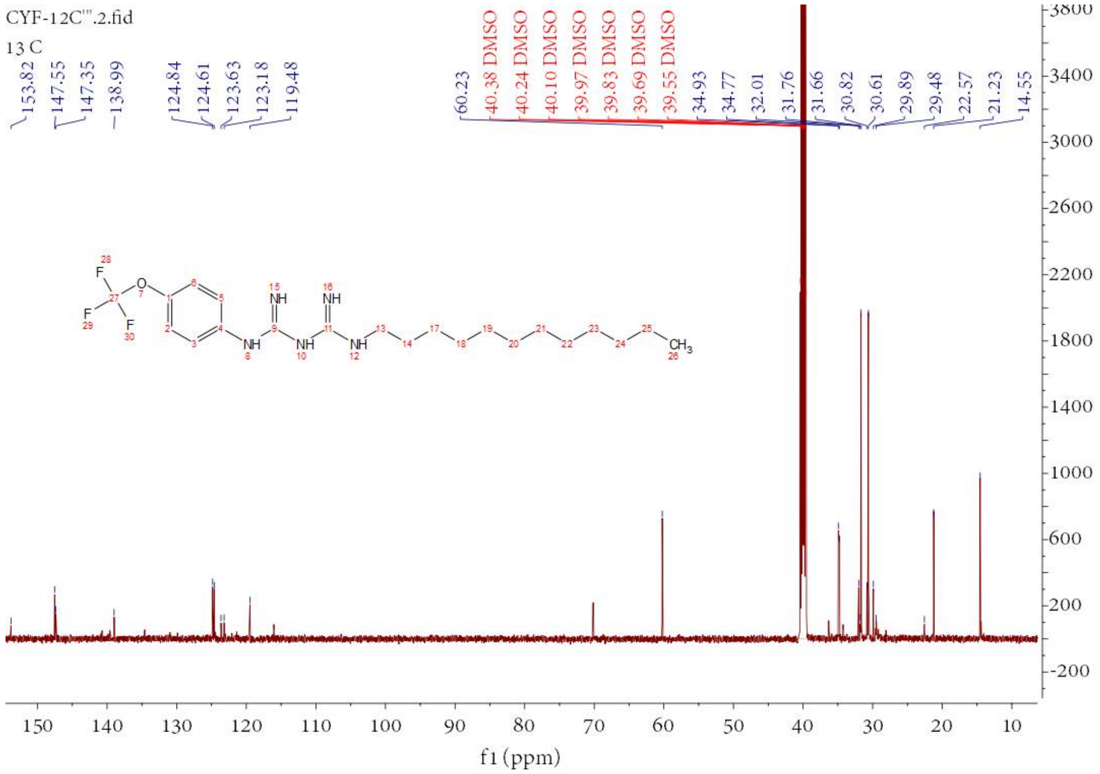

Figure S2. HPLC chromatograms of target derivatives

2C

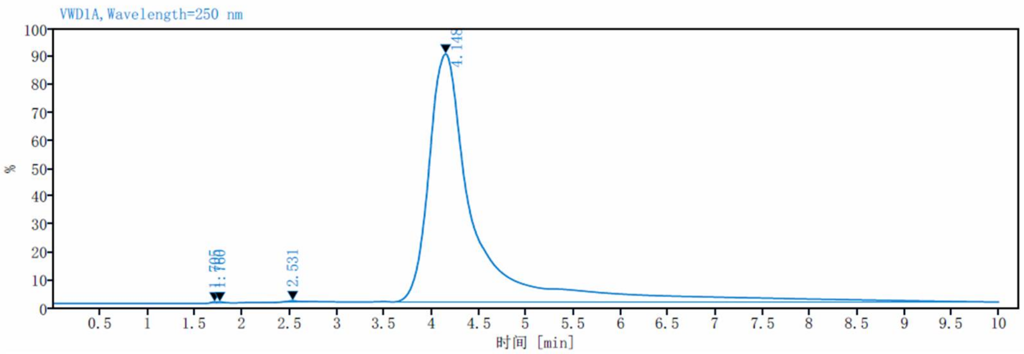

Peak	Retention time	Peak area	Peak area %	Peak width benchmark
2C	**4.148**	11698. 395	99. 74	6. 409

3C

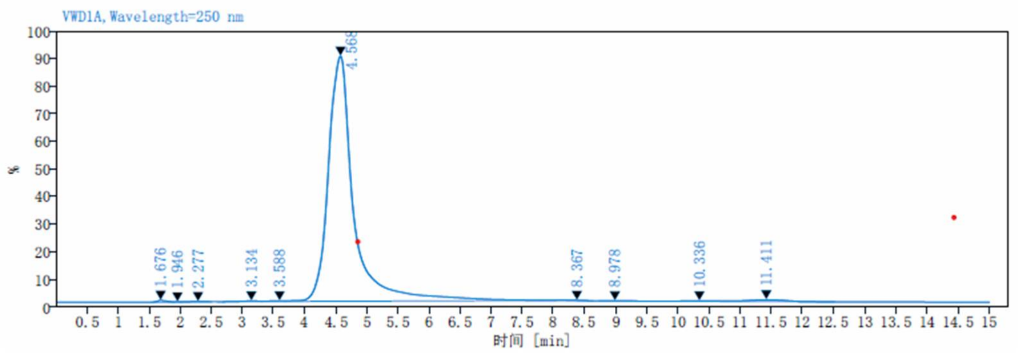

Peak	Retention time	Peak area	Peak area %	Peak width benchmark
3C	**4.568**	92415. 794	97. 75	4. 485

4C

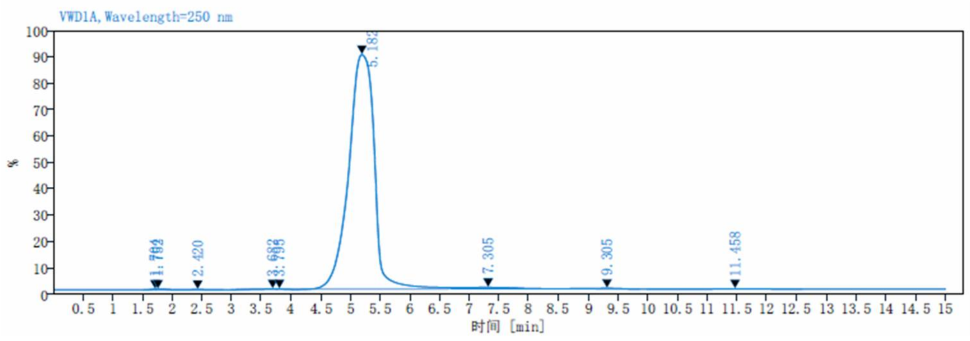

Peak	Retention time	Peak area	Peak area %	Peak width benchmark
4C	**5.182**	101576. 237	97. 52	2.821

5C

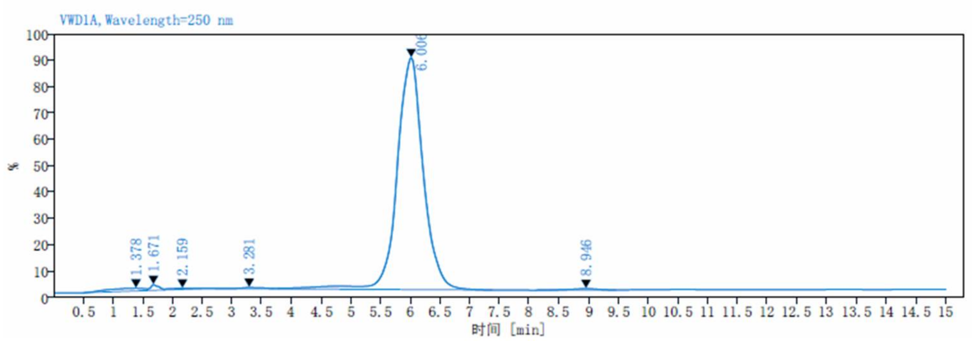

Peak	Retention time	Peak area	Peak area %	Peak width benchmark
**5C**	**6.006**	55428. 223	96. 17	0. 472

6C

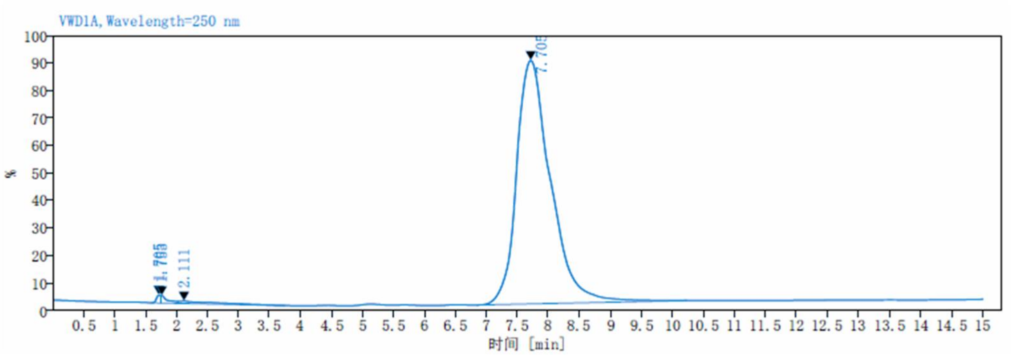

Peak	Retention time	Peak area	Peak area %	Peak width benchmark
**6C**	**7.705**	10060. 421	97. 94	3. 392

7C

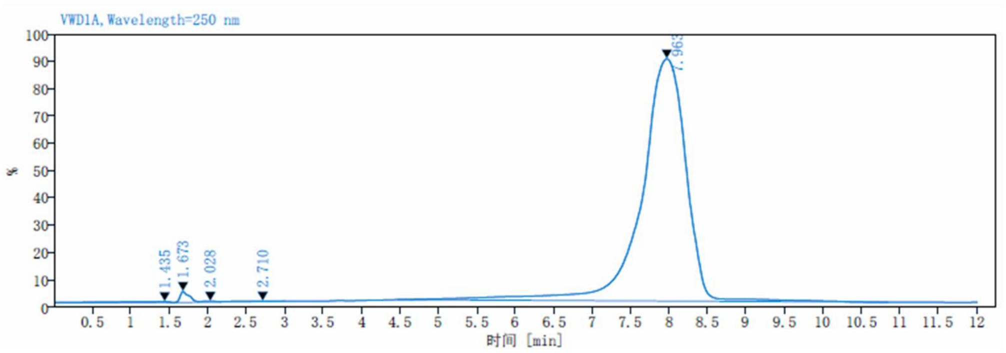

Peak	Retention time	Peak height	Peak area	Peak area %	Peak width benchmark
**7C**	**7.963**	40355.526	38772.442	98.30	6.380

8C

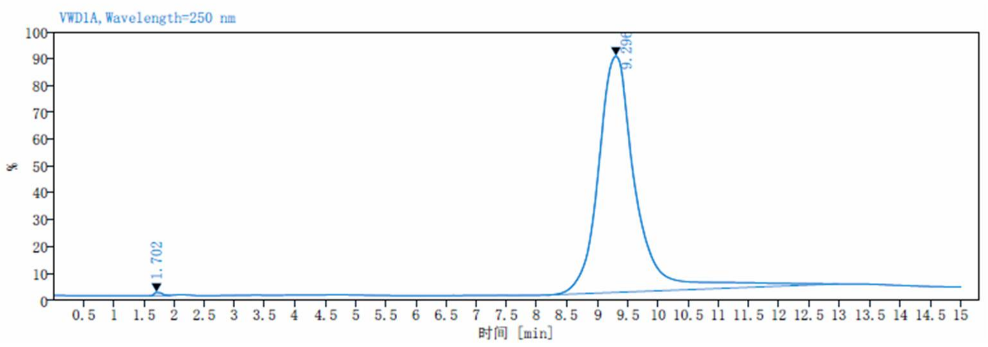

Peak	Retention time	Peak area	Peak area %	Peak width benchmark
**8C**	**9.296**	11934. 770	99. 70	4. 822

9C

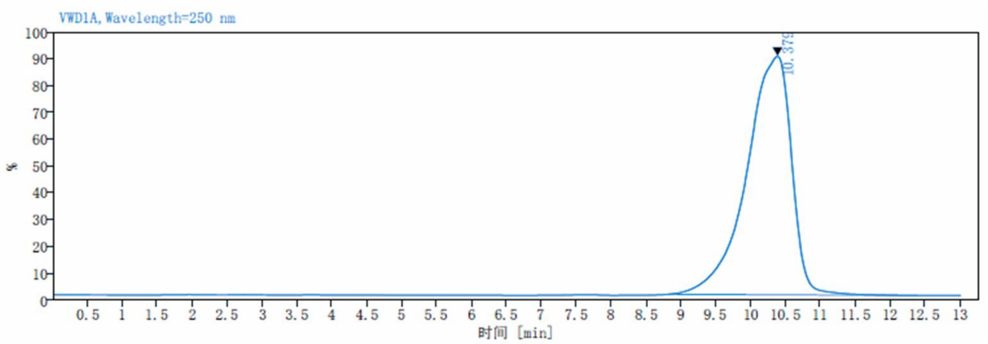

Peak	Retention time	Peak area	Peak area %	Peak width benchmark
**9C**	**10.379**	29408. 988	100. 00	3. 567

10C

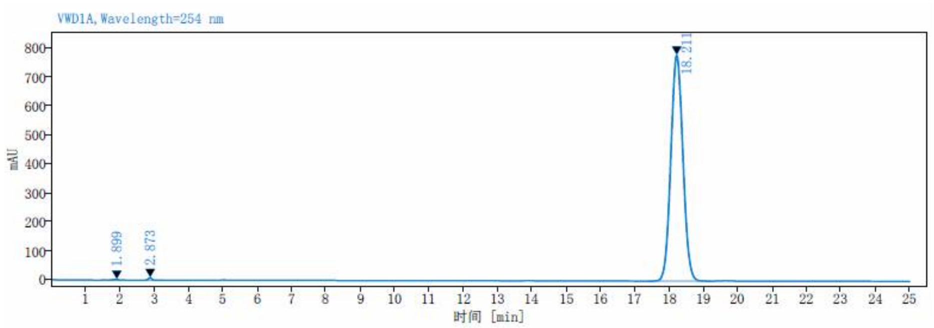

Peak	Retention time	Peak area	Peak area %	Peak width benchmark
**10C**	**18.211**	19107. 9894	99. 53	1. 827

12C

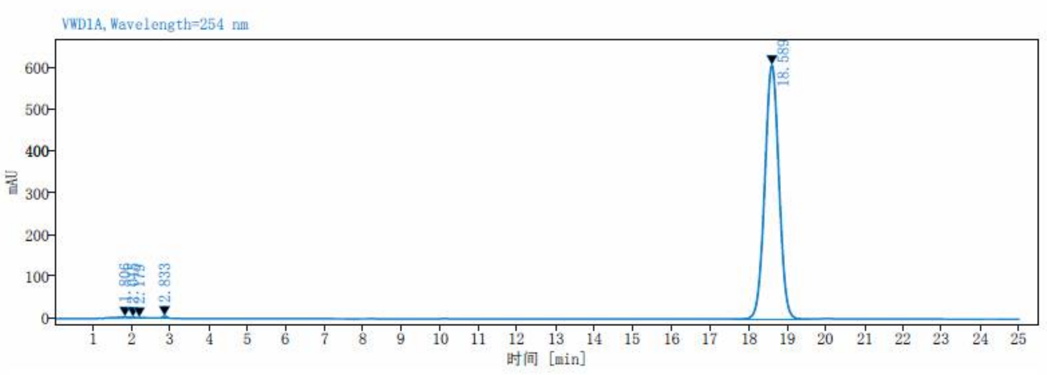

**Peak**	**Retention time**	**Peak area**	**Peak area %**	**Peak width benchmark**
**12C**	**18.589**	15471.4436	98.76	2.000

Figure S3. High Resolution Mass spectra of target derivatives

2C

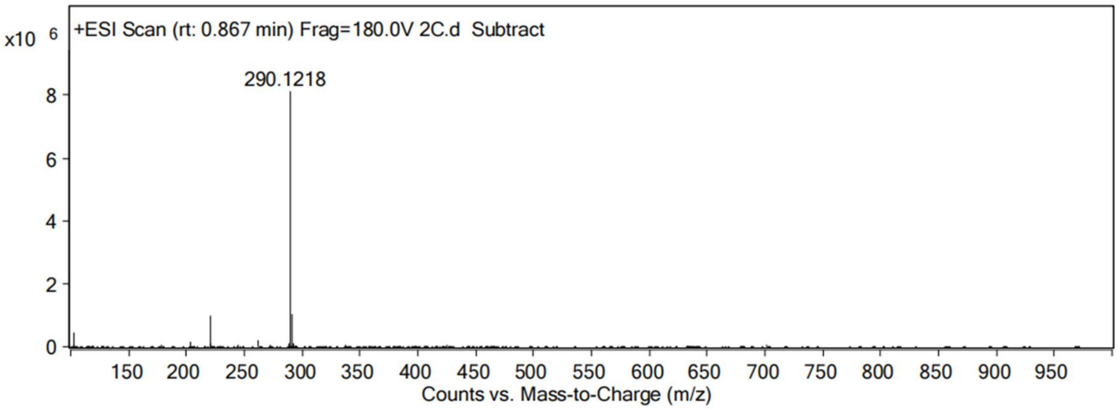

3C

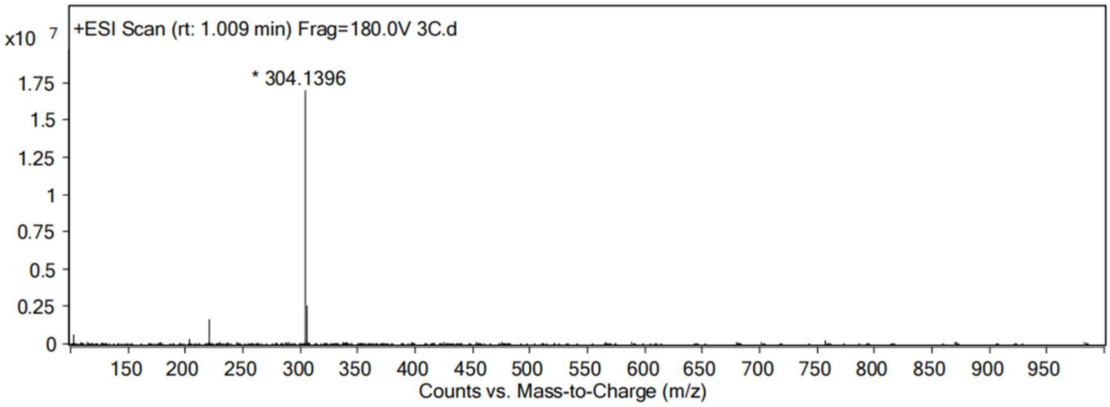

4C

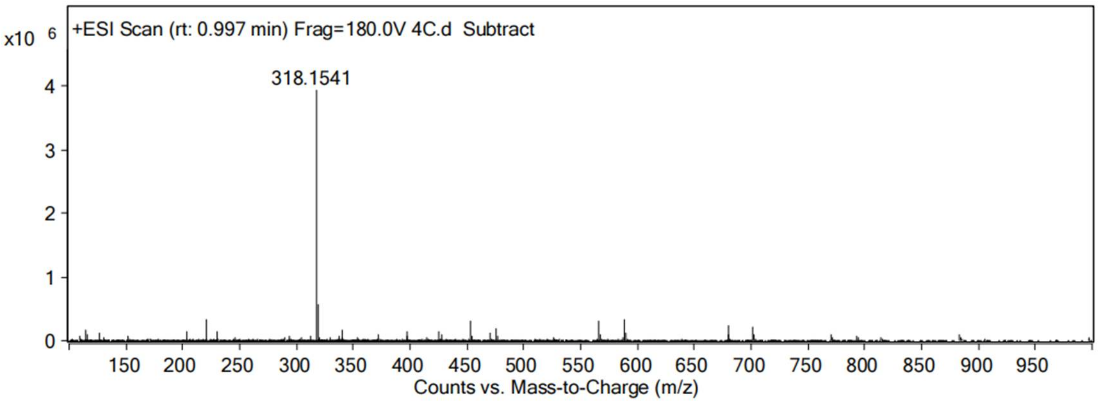

5C

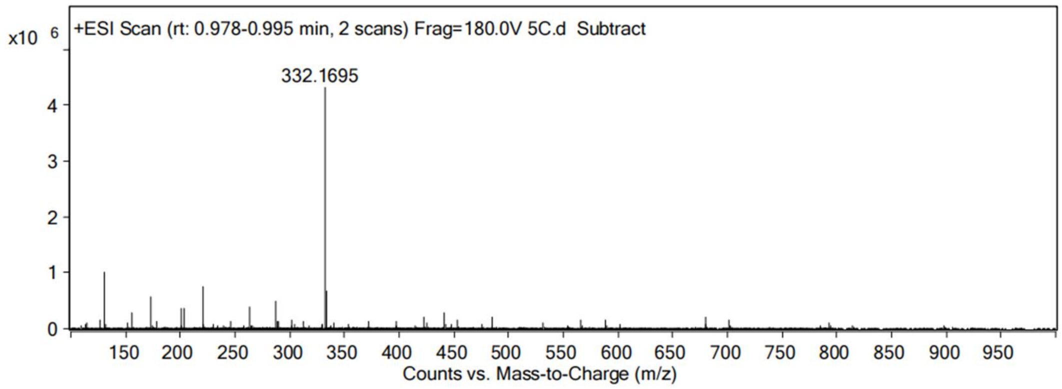
+ESI Scan (rt: 0.978-0.995 min, 2 scans) Frag=180.0V 5C.d Subtract
x10 6
332.1695
Counts vs. Mass-to-Charge (m/z)

6C

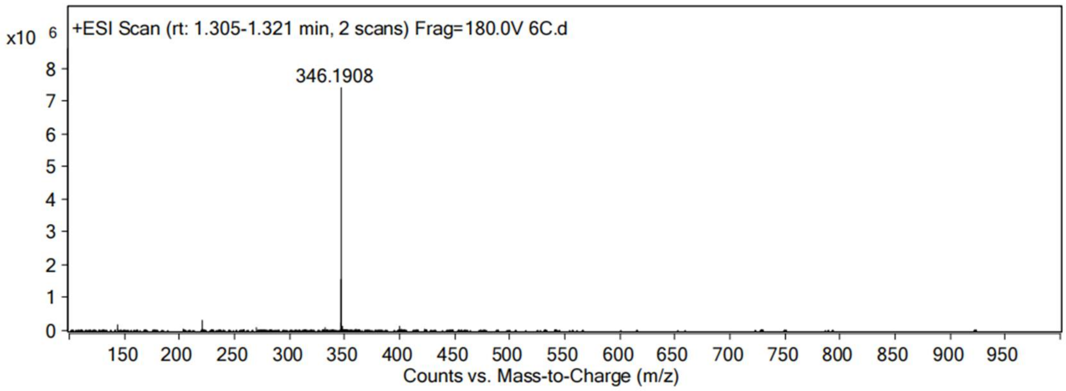
+ESI Scan (rt: 1.305-1.321 min, 2 scans) Frag=180.0V 6C.d
x10 6
346.1908
Counts vs. Mass-to-Charge (m/z)

7C

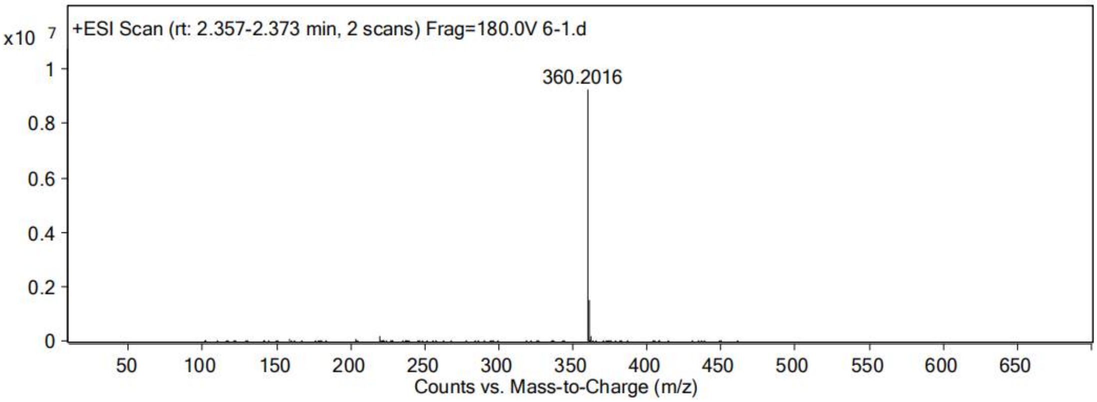
+ESI Scan (rt: 2.357-2.373 min, 2 scans) Frag=180.0V 6-1.d
x10 7
360.2016
Counts vs. Mass-to-Charge (m/z)

8C

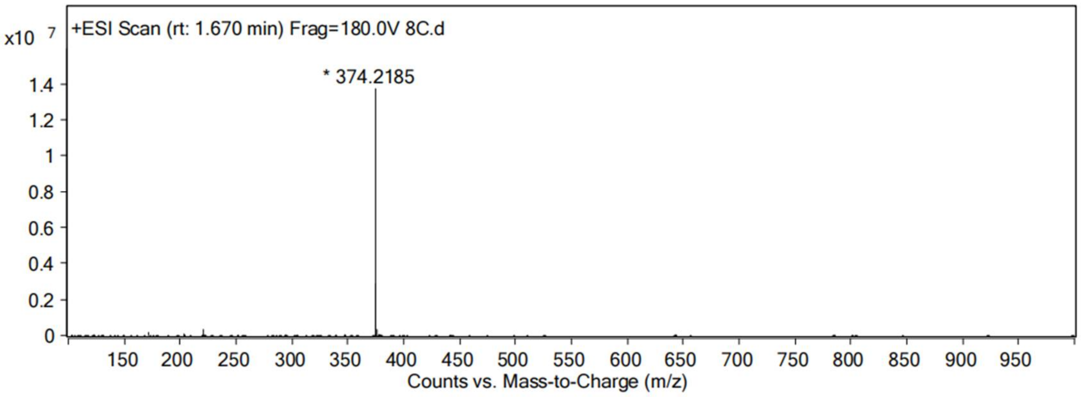

x10 7
+ESI Scan (rt: 1.670 min) Frag=180.0V 8C.d
* 374.2185
Counts vs. Mass-to-Charge (m/z)

9C

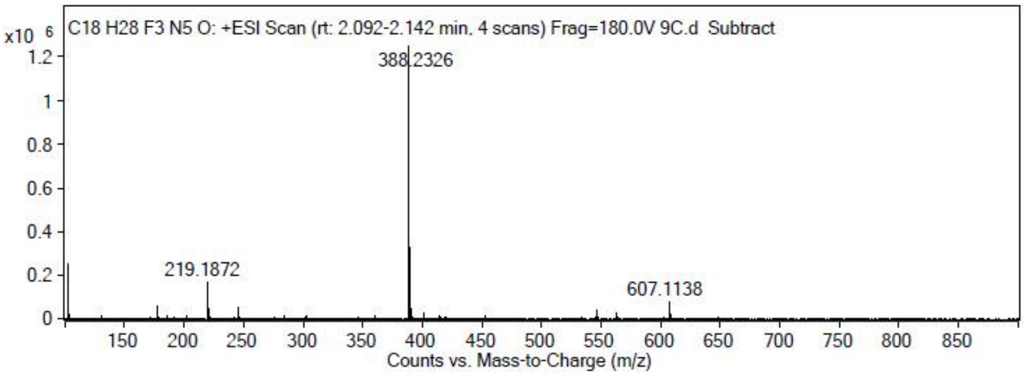

x10 6
C18 H28 F3 N5 O: +ESI Scan (rt: 2.092-2.142 min, 4 scans) Frag=180.0V 9C.d Subtract
388.2326
219.1872
607.1138
Counts vs. Mass-to-Charge (m/z)

10C

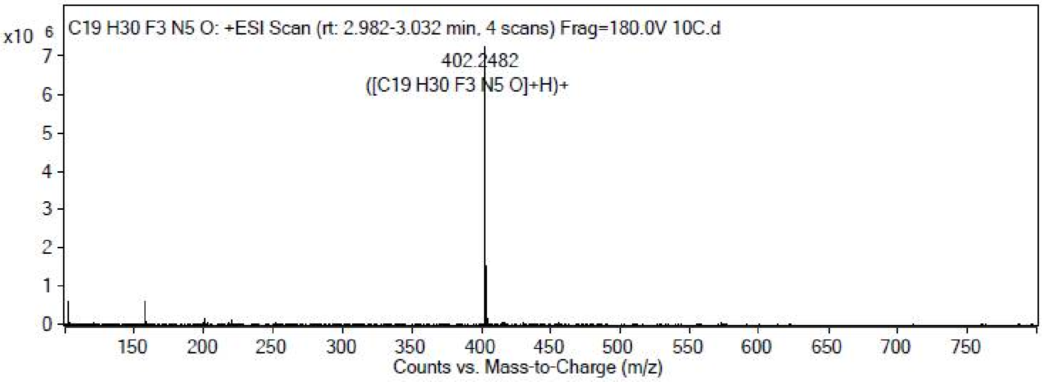

x10 6
C19 H30 F3 N5 O: +ESI Scan (rt: 2.982-3.032 min, 4 scans) Frag=180.0V 10C.d
402.2482
([C19 H30 F3 N5 O]+H)+
Counts vs. Mass-to-Charge (m/z)

12C

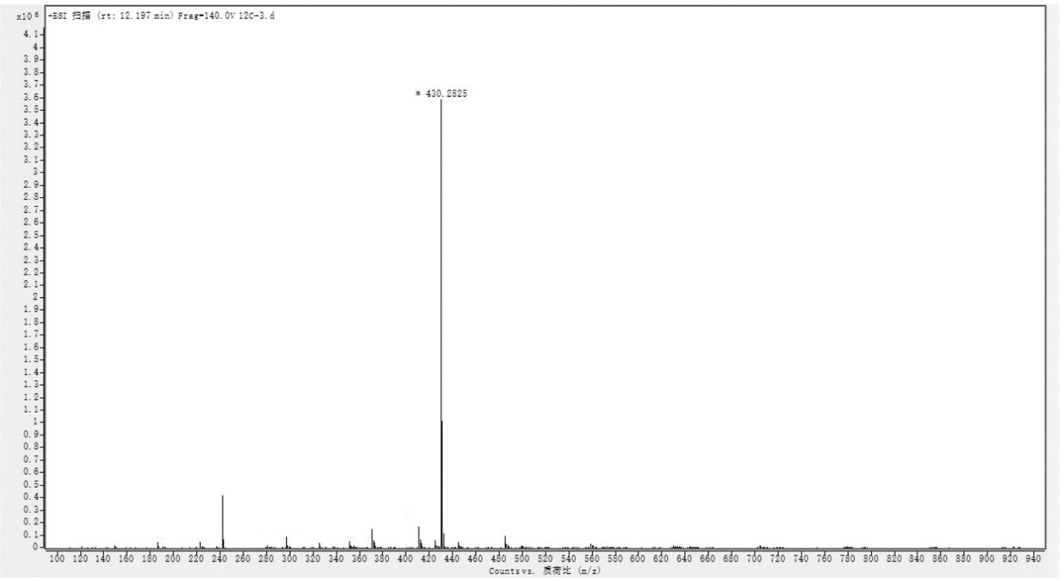
x10 6
+ESI 扫描 (rt: 12.197 min) Frag=140.0V 12C-3.d
* 430.2825
Counts vs. 质荷比 (m/z)

Figure S4. **Clonogenic assay**

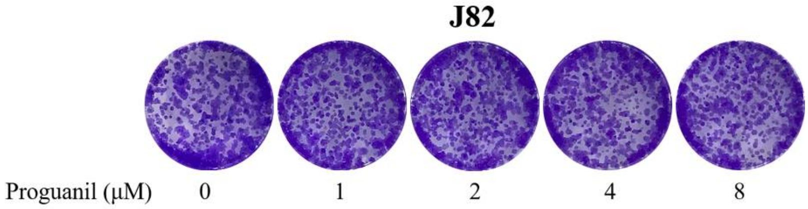

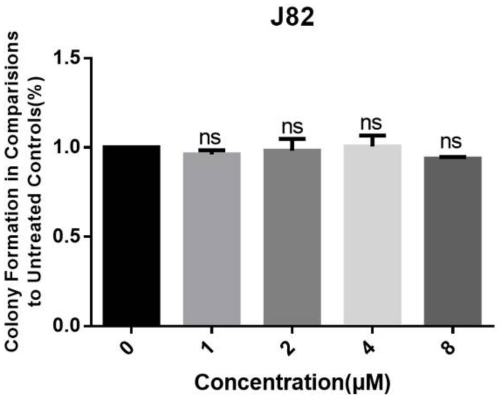

Effect of proguanil on the colony formation of J82 cell line. (a) J82 cell line was treated with proguanil at a concentration of 0-8μM, and counted with crystal violet staining after 7 days. (b) Quantification of (a) experiment. Use area scan detection at 550nm wavelength. The results are the mean ± SD of 3 independent experiments. *p<0.05, **p <0.01, and ***p<0.001 versus control (Student's t-test). SD, standard deviation.
